# The Tissue-Engineered Vascular Graft—Past, Present, and Future

**DOI:** 10.1089/ten.teb.2015.0100

**Published:** 2015-10-07

**Authors:** Samand Pashneh-Tala, Sheila MacNeil, Frederik Claeyssens

**Affiliations:** Department of Materials Science and Engineering, Kroto Research Institute, University of Sheffield, Broad Lane, Sheffield, United Kingdom.

## Abstract

Cardiovascular disease is the leading cause of death worldwide, with this trend predicted to continue for the foreseeable future. Common disorders are associated with the stenosis or occlusion of blood vessels. The preferred treatment for the long-term revascularization of occluded vessels is surgery utilizing vascular grafts, such as coronary artery bypass grafting and peripheral artery bypass grafting. Currently, autologous vessels such as the saphenous vein and internal thoracic artery represent the gold standard grafts for small-diameter vessels (<6 mm), outperforming synthetic alternatives. However, these vessels are of limited availability, require invasive harvest, and are often unsuitable for use. To address this, the development of a tissue-engineered vascular graft (TEVG) has been rigorously pursued. This article reviews the current state of the art of TEVGs. The various approaches being explored to generate TEVGs are described, including scaffold-based methods (using synthetic and natural polymers), the use of decellularized natural matrices, and tissue self-assembly processes, with the results of various *in vivo* studies, including clinical trials, highlighted. A discussion of the key areas for further investigation, including graft cell source, mechanical properties, hemodynamics, integration, and assessment in animal models, is then presented.

## Introduction

Cardiovascular disease is the number one cause of death globally.^1^ Disorders are often associated with the narrowing or blockage of blood vessels leading to reduced blood flow and tissue damage due to inadequate nutrient supply. Common presentations are coronary heart disease, cerebrovascular disease, peripheral arterial disease, and deep vein thrombosis. It is predicted that the annual incidence of cardiovascular disease-related mortalities will rise to 23.3 million worldwide by 2030.^2^

Treatments for cardiovascular disease range from dietary and lifestyle modification to pharmaceutical and surgical intervention.^3^ When required, vascular surgery may involve endovascular procedures such as angioplasty, stent insertion, or atherectomy to widen a stenosed vessel or remove the obstruction. Alternatively, a vascular graft may be used to replace or bypass a damaged or occluded vessel. Despite the advances in endovascular surgery and its increased popularity over recent decades, vascular bypass grafting remains commonplace and is believed to be the optimal choice for patients requiring long-term revascularization solutions (life expectancy >2 years).^4–8^ Around 400,000 coronary artery bypass grafting (CABG) procedures are performed each year in the United States alone.^9^

Currently, the favored conduits for vascular grafting are autologous arteries or veins. Although the use of arteries, such as the internal thoracic artery (ITA) or radial artery, is associated with superior patency,^10–13^ it is the saphenous vein (SV) that is the most commonly used autograft vessel.^14^ This is due to the limited availability of arteries and the more severe complications associated with their removal compared with veins. Despite representing the gold standard, patency rates for SV grafting remain limited with both CABG and femoropopliteal (fem-pop) bypass grafts showing failure rates of around 50% at 10 years.^14,15^ Additionally, autologous vessels have limited availability, may be of poor quality, and their extraction causes donor site morbidity.^5,15–17^

**FIG. 1.**
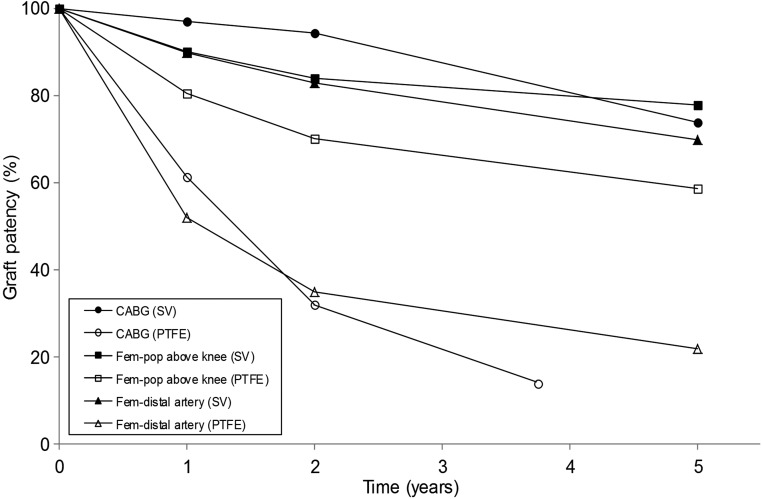
Patency rates for small-diameter vascular bypass procedures using the saphenous vein (SV) and polytetrafluoroethylene (PTFE) conduits (data for coronary artery bypass grafting [CABG] using PTFE conduits were only available up to 45 months).^14,15,20–28,32,47^

Synthetic vascular grafts are also available as an alternative to autologous vessels. These grafts have demonstrated satisfactory long-term results when used in large-diameter arteries (>8 mm), such as in aortoiliac substitutes where patency is around 90%,^18^ and in medium-diameter arteries (6–8 mm), such as carotid or common femoral artery replacements.^19^ In small-diameter vessels (<6 mm), however, synthetic grafts are of limited use due to poor patency rates. These vessels include the coronary arteries, infrainguinal arteries (below the inguinal ligament), and infrageniculate arteries (below the knee). Autologous vessels have proved superior to synthetic grafts for these installations (Fig. 1). In CABG, the use of polytetrafluoroethylene (PTFE) conduits resulted in 1-year patency rates of ∼60% compared with over 95% when using the SV. After 2 years, the patency of PTFE conduits declined to just 32%, whereas SV grafts remained above 90%.^20–23^ In above the knee fem-pop bypass, results have shown PTFE graft patency rates of ∼59% at 5 years compared with ∼78% when using the SV.^15,24–28^ A synthetic conduit is only suggested as a choice if no other suitable autologous vessel is available.^23^ Improvements in patency have been achieved by seeding autologous endothelial cells (ECs) onto the luminal surface of synthetic grafts; however, these grafts have been unable to exceed the performance of autologous vessels.^29^

Vascular graft failures are most commonly associated with thrombosis, intimal hyperplasia, atherosclerosis, or infection. Thrombosis occurs as a result of damage to, or the absence of, ECs lining the graft lumen, leading to the adherence of blood proteins and the activation of clotting mechanisms.^30,31^ Intimal hyperplasia is caused by the migration of vascular smooth muscle cells (SMCs) from the vessel media to the intima and their proliferation and extracellular matrix (ECM) deposition. Intimal hyperplasia may occur in the graft vessel or in the native vessel around the anastomosis. There are multiple causes, including (i) compliance mismatch between the graft and native vessel; (ii) vessel diameter mismatch; (iii) damage to, or a lack of, ECs; (iv) suture line stress concentrations; (v) trauma during surgery; and (vi) hemodynamic factors causing blood flow disturbances.^32–38^ Atherosclerosis appears to be the main cause of graft failure after 1 year.^39^ Atheroma formation is associated with the same factors as in the native arteries and occurs by a similar process. Monocytes invade the vessel neointima forming macrophages and, eventually, foam cells, resulting in the development of atherosclerotic plaque.^38,40,41^ Graft infection is more common in synthetic conduits due to their susceptibility to bacterial colonization. Infections cause chronic inflammation and release toxins, which complicate graft healing and can lead to sepsis and anastomotic failure or rupture.^42–46^

Given the limitations of current vascular bypass conduits, a tissue-engineered vascular graft (TEVG) presents an attractive potential solution for the future of vascular surgery. A tissue-engineered vessel with the ability to grow, remodel, and repair *in vivo*, but without the need for autograft surgery, has clear advantages and would be of great benefit. This study will review the current state of the art in vascular graft tissue engineering, including an examination of the design requirements for a TEVG, an overview of the methods being used to produce such constructs, a discussion of the various animal and limited human trials that have taken place, and a detailed outlook on the future of the field with comments on the questions still to be answered in a number of areas.

## Design Requirements for a TEVG

As an integrated part of the vascular network, a TEVG must satisfy a number of design criteria to be fit for purpose.^19,48^ Fundamentally, the construct is a conduit for supporting the flow of blood, therefore it must withstand the pressures exerted by this flow without bursting or experiencing permanent deformation through aneurysm. The pressure drop experienced within the flow over the graft length must also be sufficiently small and the luminal surface properties must be such that thrombus formation mechanisms are not triggered. The graft should possess suitable compliance to prevent the formation of high stresses around the anastomosis and be of a geometry that does not induce certain, undesirable, flow characteristics as both of these factors have been associated with failures in current bypass solutions.^32,33,37,49–51^ The graft should also be noncytotoxic and should not trigger a negative immunogenic response, such as chronic inflammation, complement cascade initiation, or activation of the adaptive immune system. Additionally, from a clinical product perspective, a TEVG should be suitable for implantation; with kink resistance and the ability to be handled, manipulated, and sutured; and be able to be mass produced in a range of lengths, quality controlled, stored, and shipped at an economically viable cost. Ultimately, the graft should be able to grow, remodel, and self-repair *in vivo*.

## Techniques for Manufacturing a TEVG

The first tissue-engineered blood vessel construct was actually produced in the mid-1980s by Weinberg and Bell.^52^ Bovine ECs, fibroblasts, and SMCs were cocultured in a collagen matrix and then shaped into tubes. Although tissue architectures analogous to natural blood vessels were achieved, the constructs required the support of a Dacron mesh and their mechanical properties were poor.

Since then, a number of different approaches have been taken to produce a clinically viable TEVG. Although these vary widely in terms of materials, manufacturing methods, cell source, and culture protocol, they can be broadly categorized into scaffold-based methods using synthetic or natural materials; decellularized natural matrix techniques and self-assembly processes.

### Scaffold-based methods

**FIG. 2.**
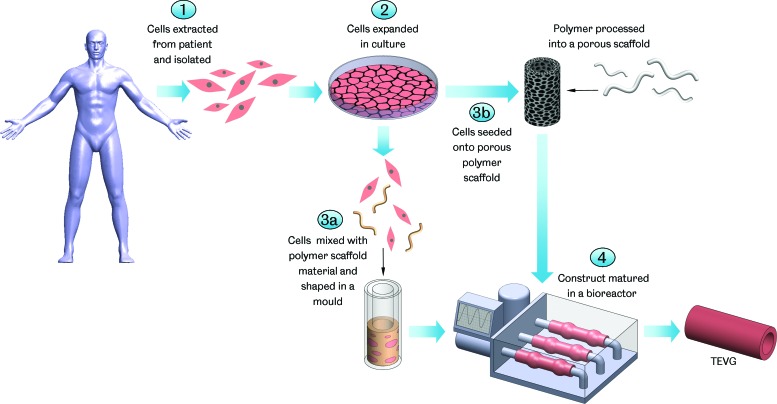
Scaffold-based tissue-engineered vascular graft (TEVG) manufacture. Cells are harvested from the patient and the required types isolated and expanded *in vitro*. The cells are then mixed with a scaffold-forming material, such as collagen or fibrin, and shaped in a tubular mold or seeded onto a porous polymer scaffold. The construct is then cultured in a bioreactor and may be conditioned to develop suitable mechanical properties for use as a TEVG. Color images available online at www.liebertpub.com/teb

Cells in culture are unable to organize themselves into complex three-dimensional structures; therefore, the use of a scaffold to provide a template of the required construct is a popular approach in tissue engineering (Fig. 2). Vascular tissue engineering has seen the use of scaffolds made from a range of synthetic and natural materials and manufactured using a number of different techniques.

#### Synthetic polymers

To date, the most extensive clinical trial of a TEVG has involved a construct produced using a synthetic polymer scaffold. The group of Shin'oka has developed a vascular graft for use in the treatment of cardiovascular disorders in children. Such disorders are particularly challenging, from a clinical perspective, often requiring multiple interventions as the child matures.^53^ A tissue-engineered graft solution with the potential to grow, remodel, and repair *in vivo* is therefore particularly suited to use in children. Using a porous scaffold produced from a degradable copolymer mesh of poly-L-lactide (PLLA) and poly-ɛ-caprolactone (PCL), reinforced with polyglycolide (PGA), vascular grafts for use as extracardiac cavopulmonary conduits to correct single ventricular physiology have been examined in 25 patients for up to 7 years.^54^ These grafts were produced by culturing autologous bone marrow-derived mononuclear cells (BM-MNCs), extracted from the anterior superior iliac spine, on the scaffolds *in vitro* before implantation. All grafts remained patent with no rupture, aneurysm formation, infection, or ectopic calcification reported, although angioplasty was required in a small number of cases to retain patency.^55^ Four patients died from nongraft-related issues during the trial. Explant examination showed complete degradation of the scaffold material and the formation of recognizable vascular tissue with a wall of SMCs and a lumenal EC layer. It was noted at late-term follow-up that 40% of patients did not require daily medication. This is considerably lower than patients receiving similar procedures using synthetic vascular grafts, which often require long-term anticoagulation or antiplatelet therapy.^56,57^ Although the success of this trial is encouraging, it must be recognized that the TEVGs constructed were of a large internal diameter (12–24 mm) and were implanted in a high-flow low-pressure system. Therefore, the success cannot be expected to easily translate into small-diameter constructs under high-pressure arterial flow. Shinoka and colleagues are attempting to adapt their approach to produce a TEVG suitable for such use, although the focus remains on potential utilization in pediatrics.^58^

With the clear aim of developing small-diameter TEVGs for use in arterial flow, Niklason and Langer have shown considerable success using a synthetic polymer scaffold-based approach. This research group pioneered the development of bioreactor systems for generating TEVGs *in vitro*, using biomimetic mechanical stimulation.^59^ It has been well established that mechanical stimulation is important in tissue engineering, having a direct impact on cell function and fate. Niklason's bioreactor design utilizes a distensible silicone tube, which carries a pulsatile flow of culture medium. When a synthetic polymer scaffold seeded with cells is placed around this tube, it is subjected to physiologically relevant strains. *In vitro* work using PGA or PGA and poly(lactide-co-glycolide) (PLGA) scaffolds seeded with bovine SMCs and ECs showed that ECM formation and vessel strength were increased by the application of mechanical stimulation compared with statically cultured controls.^59,60^ Pulsatile flow was applied for 8 weeks at 2.75 Hz (165 beats per minute), imparting a 5% radial distension, in an effort to mimic fetal development in large animals. Vessel burst pressures of 2150 ± 709 mmHg were achieved, exceeding those reported for the human SV (SV average burst pressure is 2134 ± 284 mmHg^61–65^), and vessel architectures and compliance were also comparable with natural vasculature.^66^ A subsequent *in vivo* investigation, using autologous SMCs and ECs, showed that one of these engineered vessels remained patent for up to 4 weeks when implanted in a Yucatan minipig as an SV graft with no evidence of stenosis or dilation.

As an interesting progression, Niklason and colleagues then integrated a decellularization step into their TEVG production process. Using decellularization allows nonautologous cells to be employed in producing the vessel structure during bioreactor culture on the polymer scaffold. These cells are then removed using a treatment of enzymes and detergents, leaving behind only their ECM onto which autologous ECs can then be seeded, shortly before implantation. This strategy largely decouples the TEVG manufacturing process from the recipient, potentially providing an off-the-shelf graft solution. A number of *in vivo* studies, in small and large animals, have been conducted to explore the efficacy of using an *in vitro*-derived and then decellularized graft. In nude mice, decellularized vessels derived from human SMCs were implanted as aortic interposition grafts for 6 weeks and showed 83% patency with no deterioration of the ECM as a result of the decellularization process.^67^ In porcine and canine models, decellularized grafts derived from allogeneic SMCs and then seeded with autologous ECs exhibited 100% patency at 30 days and 1 year, respectively, when implanted in carotid artery positions.^68,69^ Additional work in canines also showed 100% patency for these grafts as coronary artery bypass conduits for 30 days.^69^ In baboons, similar constructs showed 88% patency over 6 months as arteriovenous fistulas (AVFs).^69^ In all cases, grafts showed significant remodeling after implantation and the formation of tissue comparable with the adjacent native vessels. Very little intimal hyperplasia was reported, which was particularly interesting in the porcine carotid artery bypass study, as this animal model is usually considered to demonstrate accelerated intimal hyperplasia formation.^70^ These findings have now resulted in the undertaking of pilot studies in humans with the TEVG employed as an AVF for vascular access in patients with end-stage renal disease. Studies are ongoing with the first entrants recruited in 2012 and the results keenly anticipated.^71^

Results for a number of TEVGs based on synthetic polymer scaffolds have been reported by many other researchers around the world, including successes in animal models. These studies have shown great variation in relation to the polymers employed, scaffold manufacturing methods, seeded cells, and culture protocols (Table 1). In ovine models, nonwoven PGA scaffolds seeded with autologous myofibroblasts and ECs have shown long-term patency of up to 100 weeks as pulmonary artery replacements.^72,73^ These grafts had internal diameters of 10–18 mm and integrated well with the native vasculature, developing comparable tissue structures and showing complete scaffold degradation during the study period. In Lewis rats, poly(ester urethane)urea (PEUU) scaffolds produced using electrospinning and thermally induced phase separation showed success as aortic interposition grafts for 8 weeks when seeded with rat muscle-derived stem cells or human pericytes.^74,75^

**Table 1.** Studies Toward the Development of a Synthetic Polymer Scaffold-Based TEVG

*Scaffold material and manufacturing method*	*Development level*	*Cell source*	*Comments*	*Group*
P(LA/CL) and PGA or PLLA. PGA mesh coated in additional polymers in a mold.	*In vivo* (human trial)	Autologous BM-MNCs	First human trial of a TEVG. Grafts patent for up to 7 years. Explants showed complete degradation of the scaffold. Large-diameter vessel in high-flow low-pressure system.	Shin'oka and colleagues^54,55^
PGA. Mesh sewn into a tube.	*In vivo* (porcine, canine, and baboon model—human trial in progress, results pending)	Porcine SMCs and ECs or human SMCs	Early work pioneered pulsatile flow bioreactor culture for TEVGs. Grafts derived from porcine cells showed patency up to 4 weeks in the porcine SV. Later TEVGs were decellularized after *in vitro* culture. Decellularized grafts showed patency up to 6 months in a baboon model and 1 year in a canine model. Human trials underway with grafts as AVFs.	Niklason and colleagues^60,69,71^
PEUU. Thermally induced phase separation and electrospinning.	*In vivo* (murine model)	Murine muscle-derived stem cells or human pericytes	Grafts showed patency for up to 8 weeks. Host cell invasion and good integration observed. Burst pressures estimated at ∼4000 mmHg.	Vorp and colleagues^74,75,88^
PGA and P4HB. Nonwoven PGA coated in P4HB.	*In vivo* (ovine model)	Autologous ovine ECs and fibroblasts	Graft patent up to 100 weeks as a pulmonary artery replacement (large-diameter vessel). Complete scaffold degradation observed. Graft collagen content exceeded the native vessel.	Hoerstrup and colleagues^72,73^
PGS and PCL. Porogen leaching and electrospinning.	*In vivo* (murine model)	Acellular	Patent up to 90 days in the rat aorta. Graft stress–strain response similar to the native vessel. Cell infiltration and organized elastin deposition observed.	Wang and colleagues^79,89^
PGA, P(LA/CL), and P(GA/CL). Nonwoven PGA coated in P(LA/CL) and reinforced with P(GA/CL)	*In vivo* (canine model)	Acellular	Patent for 1 year in the pulmonary artery (large-diameter vessel). Scaffold fully degraded by 6 months *in vivo*. SMC and EC layers formed. Elastin and collagen content equaled the native vessel.	Yamazaki and colleagues^76^
PGA and PLLA. Woven PGA and PLLA.	*In vivo* (canine model)	Acellular	Graft patent for up to 1 year in the carotid artery. Formation of SMC and EC layers observed. Graft collagen and elastin content increased *in vivo*.	Sawa and colleagues^78^
PU. Porogen leaching.	*In vitro*	Human SMCs	Cyclic strain increased cell proliferation, collagen content, strength, and stiffness in cultured grafts.	Santerre and colleagues^90^
PGA and PCL. Polymer sheets seeded with cells concentrically wrapped.	*In vitro*	Bovine fibroblasts, SMCs, and ECs	Significant elastin deposition observed. *In vitro*-remodeled graft showed a stress–strain response similar to native bovine arteries.	Vacanti and colleagues^91^

AVFs, arteriovenous fistulas; BM-MNCs, bone marrow-derived mononuclear cells; ECs, endothelial cells; P4HB, poly-4-hydroxybutyrate; P(GA/CL), poly(glycolide/caprolactone); P(LA/CL), poly(lactide/caprolactone); PCL, poly-ɛ-caprolactone; PEUU, poly(ester urethane)urea; PGA, polyglycolide; PGS, poly(glycerol sebacate); PLLA, poly-L-lactide; PU, polyurethane; SMCs, smooth muscle cells; SV, saphenous vein; TEVG, tissue-engineered vascular graft.

The use of completely acellular synthetic polymer scaffolds as TEVGs is also being explored. Using acellular scaffolds eliminates the need for *in vitro* cell culture and instead focuses on encouraging rapid host cell invasion and scaffold remodeling, after implantation, through scaffold architecture and surface chemistry. In canine models, a scaffold produced from a composite of nonwoven PGA, poly(lactide/caprolactone) [P(LA/CL)], and poly(glycolide/caprolactone) [P(GA/CL)] exhibited patency for up to 12 months as a pulmonary artery replacement.^76^ Explants showed scaffold degradation and the formation of SMC and EC layers with ECM contents similar to the native tissue. Similar works showed patency for up to 12 months for nonwoven PLLA and PGA scaffolds and 8 weeks for electrospun PCL and polyurethane (PU) scaffolds when implanted as canine carotid artery and femoral artery interposition grafts, respectively.^77,78^ Additionally, studies in murine models have shown noteworthy results with grafts produced from electrospun PCL or poly(glycerol sebacate) (PGS) and PCL exhibiting host remodeling, ECM deposition, and native tissue structures when implanted in arterial positions.^79,80^

The use of synthetic polymer scaffolds is the most widely investigated method for producing a TEVG and has yielded significant successes. The relatively low expense of producing synthetic polymer scaffolds coupled with the ability to tune various properties associated with them has been key to their extensive use and offers great potential for the future. The mechanical properties of these scaffolds, along with their degradation rate and topography, have all been shown to influence the development of TEVGs.^75,79,81–88^ Long production times, including extended *in vitro* culture steps, present a large potential barrier to the clinical application of TEVGs based on synthetic polymer scaffolds. However, the recent use of decellularization protocols, following *in vitro* culture, to largely move TEVG production offline and the potential of acellular scaffold-only grafts both offer hope for the future.^69,76–80^

#### Natural polymers

A number of different naturally derived polymers have been employed to generate scaffolds for use in TEVG production (Table 2). Significant successes have been seen using fibrin as a scaffold material. This material can be produced from polymerized fibrinogen isolated from a patient's own blood plasma.^92^ The group of Tranquillo used fibrin gel to entrap human dermal fibroblasts and produce tubes using a mold.^93^ A TEVG was then assembled by concentrically layering these tubes and allowing them to fuse together; however, after 3 weeks in culture, burst pressure values were just 543 ± 77 mmHg, well below those for natural vessels. Similar to synthetic polymer scaffolds, the application of mechanical stimulation improved the vessels' mechanical strength.^94^ *In vitro* culture in a perfusion bioreactor and applying cyclic strain and lumenal, ablumenal, and transmural flow generated a TEVG with a burst pressure of 1542 ± 188 mmHg and comparable compliance to natural vasculature. In a similar approach to Niklason's group, Tranquillo and colleagues have also recently reported on the use of decellularization in their production of TEVGs.^95^ Fibrin-based grafts were cultured *in vitro* using ovine dermal fibroblasts and then decellularized. These acellular grafts exhibited comparable compliance to human vasculature and burst pressures exceeding those of the human SV. When implanted in the femoral artery of an ovine model, these grafts remained patent for up to 24 weeks with no occlusion, dilation, or mineralization reported, representing the first long-term function of a natural polymer scaffold-based TEVG in the artery of a large animal model. Explants demonstrated considerable remodeling with complete graft cellularization and increased collagen and elastin content.

**Table 2.** Studies Toward the Development of a Natural Polymer Scaffold-Based TEVG

*Scaffold material and manufacturing method*	*Development level*	*Cell source*	*Comments*	*Group*
Fibrin. Gelled with encapsulated cells.	*In vivo* (ovine model)	Ovine dermal fibroblasts	Fibrin-based TEVG cultured *in vitro* then decellularized. Decellularized constructs possessed burst pressures of ∼4200 mmHg and compliance similar to natural vessels. Grafts remained patent for up to 24 weeks in the femoral artery and completely recellularized.	Tranquillo and colleagues^93–95^
Fibrin. Gelled with encapsulated cells.	*In vivo* (ovine model)	Ovine vascular SMCs, bone marrow smooth muscle progenitor cells, and ECs	Patent for up to 15 weeks in the jugular vein. Grafts integrated well with the native vessel and remodeled to 25% the strength of the ovine aorta. Progenitor cell-based TEVGs were stronger than those derived from vascular SMCs and produced greater elastin *in vivo*.	Andreadis and colleagues^96,98^
Silk fibroin. Gel spun into a tube.	*In vivo* (murine model)	Acellular	Patent for up to 4 weeks in the rat aorta. Graft invasion by host SMCs and ECs observed.	Kaplan and colleagues^100^
Silk fibroin. Woven into a tube.	*In vivo* (murine model)	Acellular	Patent for up to 1 year in the rat aorta. SMC and EC invasion observed at 12 weeks. Fibroin content reduced by 48 weeks, while collagen content increased.	Sata and colleagues^101^
Silk fibroin. Electrospun and then coated with collagen.	*In vitro*	NIH/3T3 fibroblasts	Cells adhered and proliferated on the scaffold. Construct strength was below that of natural vessels (∼900 mmHg).	Mantovani and colleagues^103^
Collagen. Gelled with encapsulated cells.	*In vitro*	Porcine SMCs and ECs	Cell proliferation and collagen remodeling observed over 7 days. Very low burst pressures achieved (∼18 mmHg).	Mantovani and colleagues^116^
Collagen. Gelled with encapsulated cells.	*In vitro*	Murine aortic SMCs	Construct strength improved by increased collagen deposition as a result of mechanical stimulation. Burst pressures remained well below those of natural blood vessels.	Nerem and colleagues^109^
Collagen and elastin. Freeze-dried then cross-linked.	*In vitro*	Human umbilical vein SMCs	Construct strength improved by mechanical stimulation. Stress–strain curve partially matched native vessels.	Feijen and colleagues^111,117^
Chitosan and gelatin. Knitted chitosan tube dipped in gelatin and freeze-dried	*In vitro*	Murine vascular SMCs	Burst pressures of 4000 mmHg achieved. Suture retention strengths also exceeded the ovine carotid artery.	Zhang and colleagues^113^

Additionally, Andreadis and colleagues have also examined a fibrin-based TEVG in an ovine model. In this study, fibrin tubes with entrapped vascular SMCs were implanted as vein interposition grafts in lambs.^96^ The lumenal surface of these TEVGs was seeded with ECs before implantation and they showed patency for up to 15 weeks. Examination of explants showed that *in vivo* remodeling increased graft mechanical strength; however, this reached only 25% of that of the native aorta. Altering fibrinogen concentrations and using bone marrow-derived smooth muscle progenitor cells were demonstrated to produce stronger vessels, although this design of TEVG still remains to be tested under atrial flow.^97,98^ Recently, hypoxia coupled with insulin supplementation was also shown to improve the strength of fibrin-based TEVGs by enhancing collagen deposition in the entrapped cells.^99^

Silk-derived fibroin also has potential as a scaffold material for TEVGs. It offers tailorable mechanical properties, slow degradation *in vivo*, and is compatible with a number of manufacturing processes.^100^ *In vitro* work, using woven and electrospun fibroin scaffolds, has shown acceptable biocompatibility and adherence using a range of vascular cell types.^100–102^ In subsequent studies in rats, as abdominal aorta replacements, acellular fibroin scaffolds showed cell invasion by SMCs and ECs and vascular tissue formation. Patency rates of 85% at 12 months were achieved, with no thrombosis or aneurysm observed. It was also suggested that the mechanical properties of these scaffolds could be improved to better emulate those of the native vasculature by using fiber alignment techniques during the manufacturing process.^100^ Additionally, Mantovani and colleagues have constructed a scaffold comprising silk fibroin and collagen using electrospinning. Although this was shown to have superior strength compared with fibroin alone, it remained weaker than natural vessels. Viscoelasticity and good cell adherence were also shown, although there are as yet no reports of *in vivo* results.^103^

Building on the early work of Weinberg and Bell,^52^ the use of collagen in TEVG scaffolds has continued. The integrity of collagen-based scaffolds has been improved by modifying fiber density and orientation, adding cross-links, and using specific shape-forming techniques.^104–108^ Mechanical stimulation during *in vitro* culture has also been used. Using a bioreactor to apply mechanical conditioning through cyclic strain was shown to improve tissue organization and significantly increase the strength of collagen gel-based TEVGs.^109,110^ The addition of elastin fibers to form a hybrid scaffold was also shown to alter the mechanical properties of engineered vessels to more closely resemble those of natural tissue, with a nonlinear, J-shaped stress–strain response.^111^ In all cases, however, the ultimate tensile strengths and burst pressures of these constructs remained well below those of native vessels.

The linear polysaccharide, chitosan, has also been considered for the production of TEVG scaffolds. Chitosan is a derivative of chitin and is similar in structure to glycosaminoglycans, which are a common ECM element.^112^ Porous structures can easily be fabricated from chitosan using freezing or lyophilization techniques and, *in vivo*, the material is slowly degraded by lysozyme with little foreign body reaction. Using a mesh of knitted chitosan fibers, coated in a chitosan and gelatin solution and then freeze-dehydrated, a porous scaffold was produced with a burst pressure of 4000 mmHg and suture retention strength of 4.4 N.^113^ Both of these values exceed those of the native vessel they were compared with (ovine carotid artery). The scaffold also showed acceptable cell adhesion and proliferation over 2 days using rabbit vascular SMCs. Although this report is promising in terms of mechanical performance, continued work using this scaffold is lacking. An alternative, constructed from cross-linked and freeze-dried chitosan and collagen, has also been shown to support vascular cell adhesion and proliferation and, additionally, exhibited suitable biocompatibility *in vivo* when implanted in rabbit livers.^114^ However, the scaffold mechanical properties reported in this case were inferior to native blood vessels, with an ultimate tensile strength of just 310 ± 16 kPa. A question mark clearly remains over the mechanical properties of a chitosan scaffold and also how these may change over longer periods of *in vitro* cell culture or *in vivo*.

Although advantageous in terms of cell adhesion and biocompatibility, natural polymer-based TEVGs have largely remained limited by their poor mechanical strength. Additionally, a high degree of compaction occurs in a number of natural polymer scaffolds, which produce a very dense matrix that vascular cells may have difficulty breaking down to remodel.^115^ The use of dynamic bioreactor culture to improve vessel mechanical properties has now been demonstrated.^94,96,98^ Although the use of bioreactors increases production complexity, the recent successes seen in decellularized fibrin-based TEVGs show the possibility of an off-the-shelf graft solution even if lengthy *in vitro* culture is required. This graft design has great potential for clinical utility, but is yet to be evaluated in the human circulatory system.

#### Hybrid polymer scaffolds—synthetic and natural polymers

Not all scaffold-based approaches to producing a TEVG have been based exclusively on natural or synthetic polymers. A number of researchers have developed scaffold systems that utilize a combination of both (Table 3).

**Table 3.** Studies Toward the Development of a Hybrid Polymer Scaffold-Based TEVG

*Scaffold material and manufacturing method*	*Development level*	*Cell source*	*Comments*	*Group*
P(L/D)LA and fibrin gel. Extruded polymer surrounded by fibrin gel with encapsulated cells.	*In vivo* (ovine model)	Ovine SMCs, fibroblasts, and ECs	Patent up to 6 months in the ovine carotid artery. Graft integrated well with the native vessel. Collagen and elastin deposition observed.	Jockenhoevel and colleagues^120^
PCL and collagen. Electrospun.	*In vivo* (lapine model)	Acellular	Patent for up to 1 month as rabbit aortoiliac bypass grafts. Little cell invasion or thrombosis observed.	Atala and colleagues^122^
PCL, spider silk, and chitosan. Electrospun.	*In vivo* (murine model)	Acellular	Patent up to 8 weeks in the rat aorta. Host cell invasion shown.	Zhang D and colleagues^121^
PCL and synthetic elastin. Electrospun.	*In vivo* (lapine model)	Acellular	Similar mechanical properties to the ITA demonstrated. Grafts remained patent for 1 month in the rabbit carotid artery.	Weiss and colleagues^61^
PCL and PU-collagen composite. Electrospun.	*In vivo* (canine model)	Acellular	Patent for up to 8 weeks in the canine femoral artery. A thin layer of ECs formed *in vivo*.	Zhang J and colleagues^77^
Gelatin–vinyl acetate copolymer. Electrospun.	*In vitro*	Murine SMCs	Dynamic culture conditions increased ECM deposition. Collagen and elastin content reached 70–80% that of the native rat aorta in 5 days.	Thomas and Nair^81^
PU and PEG-fibrin. Electrospun PU with seeded cells rolled up and coated in PEG-fibrin hydrogel.	*In vitro*	Murine smooth muscle progenitors	Graft stress–strain response after dynamic culture was very similar to the human coronary artery, although with lower ultimate tensile strength.	Hahn and colleagues^84^

ECM, extracellular matrix; ITA, internal thoracic artery; P(L/D)LA, poly(L/D)lactide; PEG, polyethylene glycol.

Coating of synthetic polymer scaffolds with natural polymers, in an effort to improve biocompatibility and cell adhesion, has been used extensively with collagen, fibronectin, and gelatin, all employed.^78,83,118,119^ Synthetic polymers have also been used to provide reinforcement to weaker, natural polymer scaffolds. Fibrin gels, with encapsulated ovine SMCs and fibroblasts, reinforced with a mesh of poly(L/D)lactide [P(L/D)LA] have been studied in an ovine model.^120^ Following a 21-day culture in a perfusion bioreactor, these engineered vessels were implanted as carotid artery interposition grafts and showed patency for up to 6 months with dense, although not cohesive, collagen and elastin deposition observed. In other work, a bilayered scaffold with an inner layer of recombinant human tropoelastin and an outer layer of PCL has demonstrated success in a rabbit model.^61^ These grafts possessed mechanical properties not significantly different from the human ITA in terms of compliance and burst pressure. The elastin appeared to aid cell attachment and also conferred reduced platelet adhesion. When implanted as acellular grafts in the carotid artery position, they showed 100% patency over 1 month with no dilation or thrombosis and little change in mechanical properties. Additionally, good results have been reported for acellular hybrid vessels of PCL, with PU and collagen or spiders' silk and chitosan, in arterial positions in canines and rats, respectively.^77,121^

These hybrid scaffolds may be considered as new smart biomaterials that incorporate the strength, tunability, and manufacturing control of synthetic materials with the improved biocompatibility and biochemical cues that come from natural polymer components. Therefore, the use of hybrid scaffolds has the potential to exploit the best of both synthetic and natural polymer scaffold systems to produce TEVGs. However, some of the limitations associated with using polymer scaffolds to generate a TEVG are likely to remain a factor, particularly the requirement for long periods of *in vitro* culture to generate robust constructs.

### Decellularized natural matrices

**FIG. 3.**
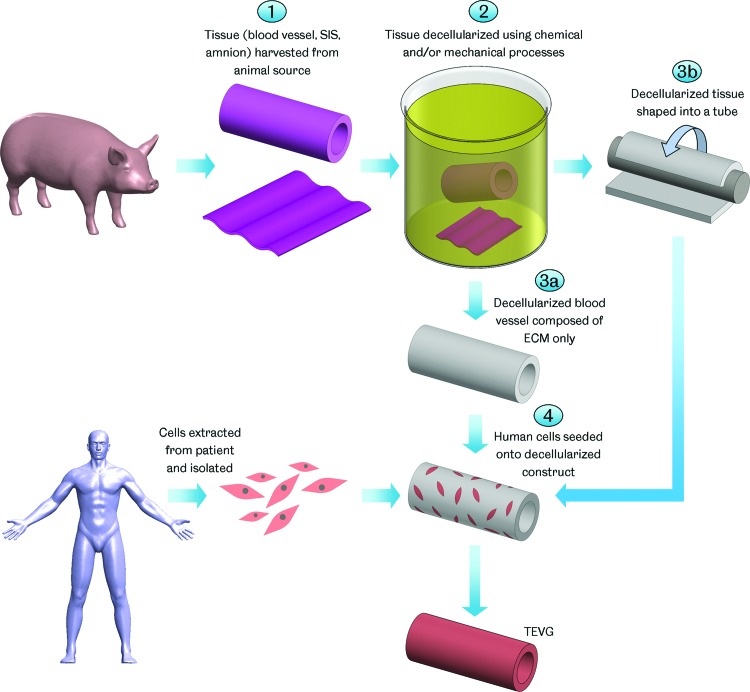
TEVG manufacture using decellularized matrices. Tissue is harvested from an animal source and decellularized using various chemical and/or mechanical processes. Where vascular tissue is decellularized, the result is a tube comprising only extracellular matrix (ECM). Decellularized nonvascular tissue, such as small intestinal submucosa (SIS) or amniotic membrane, may be shaped into a tubular construct. Cells extracted from the patient are then seeded onto the decellularized scaffold forming a TEVG after maturation. Color images available online at www.liebertpub.com/teb

The use of decellularized natural matrices in tissue engineering takes advantage of the structure and mechanical performance of natural tissue ECM while avoiding any adverse immunological reactions due to its origin. The decellularization process refers to the removal of antigenic cellular material from the tissue (Fig. 3). Decellularization may involve a variety of chemical agents, such as acids and bases, hypo/hypertonic solutions, detergents and solvents; biological agents, such as enzymes and chelating agents; and physical methods, such as agitation, pressure, and abrasion.^123^ Preservation of the ECM is intended to maintain the tissue's mechanical properties.^124,125^ A number of clinical products based on decellularized tissues, both human and animal in origin, are available for a wide range of applications, including dermal, soft tissue, cardiac, ophthalmic, and dentistry.^123^

Decellularized vascular grafts were first developed in the 1960s using animal tissue.^126^ In the years since then, a range of these grafts have been made commercially available. These include Artegraft^®^, Solcograft^®^, and ProCol^®^, which were based on decellularized bovine blood vessels, and SynerGraft^®^ model 100 which was derived from decellularized bovine ureter.^127–134^ Although these grafts have been utilized in vascular bypass surgery and as vascular access conduits, their large-scale adoption has not been seen. A number of studies, including prospective randomized trials, concluded that these decellularized xenogeneic grafts offered no clear advantage compared with alternative synthetic conduits.^135–138^ Patency rates were comparable, at best, and the probability of graft salvage in the event of complications, such as infection or pseudoaneurysm, was lower. Decellularized xenogeneic grafts also cost considerably more than synthetic grafts.

A product based on decellularized human donor veins has also been developed and commercialized for use as an AVF (SynerGraft processed human cadaver vein allograft). However, just as with decellularized xenogeneic grafts, this has not been widely adopted. Studies suggested that this graft offered no improvement in patency compared with established solutions. The decellularized human vessels appeared more resistant to infection compared with synthetic alternatives, but were more susceptible to aneurysm.^139^ Additionally, the availability of the human donor vessels required to produce this product is unpredictable and there are complex ethical and regulatory issues associated with the commercialization of such tissue.

The limited performance of commercially available decellularized vascular grafts has been suggested to be due to their lack of cellularity on implantation.^127,133^ The major failure modes observed are graft-related thrombosis, infection, and aneurysm. These may be combated by adding cells to the grafts, particularly luminal ECs, before implantation. TEVG developers have taken to exploring this strategy (Table 4). In a first in man study, a decellularized human iliac vein seeded with autologous cells has shown success when used to produce a conduit for extrahepatic portal vein obstruction bypass (meso-Rex bypass) in a pediatric case.^140^ The vein was decellularized with detergents and enzymes and then seeded with autologous bone marrow-derived ECs and SMCs *in vitro*. After 6 days of bioreactor culture, the vessel was successfully implanted in a 10-year-old girl. Patency was reported up to 2 years, although narrowing of the graft, at 9 months, required the insertion of a second section. This procedure offers potential, although only a single human implant has been reported and this was in low-pressure flow.

**Table 4.** Studies Toward the Development of a Decellularized Natural Matrix-Based TEVG

*Scaffold material and manufacturing method*	*Development level*	*Cell source*	*Comments*	*Group*
Bovine carotid artery. Decellularized.	*In vivo* (clinical experience)	Acellular	First commercialized decellularized vascular grafts. Comparing performance with established PTFE conduits showed no significant improvement in patency when used as AVFs. Salvage of decellularized grafts was more challenging after complications compared with PTFE grafts.	Sterling and colleagues^132^ Johnson and colleagues^165^
Bovine mesenteric vein. Decellularized.	*In vivo* (clinical experience)	Acellular	Poor results when used in peripheral bypass procedures. Failures associated with thrombosis and aneurysm. Some success compared with PTFE conduits when used as AVFs in high-risk patients, although data are limited.	Lawson and colleagues^128^ Davies and colleagues^130^
Bovine ureter. Decellularized.	*In vivo* (clinical experience)	Acellular	Prospective randomized trial comparing decellularized bovine ureter with PTFE conduits when used as AVFs. No significant difference in performance was found.	Chemla and Morsy^127^
Human vein. Decellularized.	*In vivo* (clinical experience)	Acellular	Compared results for decellularized human veins, cryopreserved human veins, and PTFE conduits as AVFs. Decellularized grafts showed no improvements in patency.	Kurbanov and colleagues^139^
Human iliac vein. Decellularized.	*In vivo* (human trial)	Autologous SMCs and ECs	First human trial of a decellularized vessel seeded with stem cell-derived autologous cells. Graft as an extrahepatic portal vein bypass. Patent for up to 2 years, although partial narrowing at 9 months required the addition of a second graft section.	Sumitran-Holgersson and colleagues^140^
Porcine artery. Decellularized.	*In vivo* (ovine model)	Autologous ovine ECs	Grafts showed an average patency of 4.4 months as AVFs. ECs covered 50% of the graft lumen after 6 months.	Atala and colleagues^141^
Canine carotid artery. Decellularized.	*In vivo* (canine model)	Canine bone marrow-derived SMCs and ECs	Patent for up to 8 weeks in the carotid artery. Cell-seeded grafts performed better than acellular controls. Explants showed a layered vascular wall structure with collagen and elastin deposition.	Kim and colleagues^124^
Human umbilical vein. Frozen, machined to a uniform diameter, and then decellularized.	*In vitro*	Human umbilical cord vein ECs or fibroblasts	Bioreactor culture shown to increase vessel burst pressures. Burst pressures remained below that of human arteries at ∼1200 mmHg.	Tosun and McFetridge^162^
Porcine SIS. Decellularized and shaped into a tube.	*In vivo* (canine model)	Acellular	Patent for up to 60 days in the carotid artery. Explant vessels showed increased strength due to remodeling. Burst pressures exceeded human vessels (∼5600 mmHg).	Lantz and colleagues^144,145^
Human amniotic membrane. Membrane seeded with cells and then rolled up.	*In vitro*	Human SMCs and umbilical vein ECs	Graft stress–strain response was similar to human vasculature. Rupture strengths were 71% that of the human carotid artery.	Amensag and McFetridge^149^

AVFs, arteriovenous fistulas; PTFE, polytetrafluoroethylene; SIS, small intestinal submucosa.

A variety of decellularized vessels seeded with cells have been evaluated in animal studies also. In ovine models, positive results have been reported for decellularized porcine carotid arteries as carotid artery bypass grafts and AVFs. Following seeding with endothelial progenitor cells (EPCs), these grafts showed patency up to 4.3 and 5.6 months, respectively.^141,142^ Additionally, in canines, decellularized carotid arteries (porcine and allogeneic) have shown patency up to 2 months in the carotid artery position.^124,125^

Nonvascular tissues, such as the small intestinal submucosa (SIS) and amniotic membrane, have also been decellularized and used to produce TEVGs. The SIS is a natural ECM sheet that has seen clinical application in many areas, including skin, bladder, tendon, and intestine.^143^ Early work used porcine SIS, decellularized by abrasion and sutured into tubes, to produce TEVGs. When implanted in an acellular state, these grafts showed superior patency compared with PTFE conduits over 180 days in the canine carotid artery.^144^ Host cell invasion and remodeling altered their compliance and burst pressures similar to the native vessel.^145^ Implantation in the canine aorta also showed good patency and remodeling, although the change in graft compliance was smaller.^146^ More recently, ovine SIS has been examined *in vitro* for its potential as a TEVG. When cultured as sheets under uniaxial strain, this tissue supported SMCs, differentiated from a hair follicle, which deposited collagen and elastin. The tissue also showed compliance properties similar to the native ovine carotid artery, although with lower tensile strength.^147^

The amniotic membrane is another natural ECM sheet, forming the inner layer of the placental membrane. It is covered by epithelium and contains collagen, fibronectin, and laminins and has been shown to be biocompatible and nonimmunogenic in ocular surface transplantation.^148^ TEVGs produced *in vitro* using the human amniotic membrane supported the growth and proliferation of ECs and SMCs. These constructs utilized decellularized amnion, either shaped around a mandrel and chemically cross-linked with glutaraldehyde or as a base for the culture of a cell sheet before being rolled into a tube.^149,150^ In the latter, mechanical testing of the cultured vessels after 40 days under pulsatile flow showed a J-shaped stress–strain response, indicative of soft tissue, and a rupture strength 71% that of the human carotid artery, although the elastic modulus and compliance properties differed somewhat from this vessel. In a further step, TEVGs constructed from the human amniotic membrane sutured into tubes were examined in a lamb model as interposition grafts in the jugular vein.^151^ These grafts showed 100% patency over 48 weeks with no dilation, thrombosis, or stenosis. Despite being implanted with their epithelium intact, little immune response was observed, highlighting the low immunogenicity of the amniotic membrane. It remains to be seen how grafts produced using the amniotic membrane perform under arterial flow and pressure.

The natural architecture of decellularized tissues coupled with their diverse structural and functional biomolecular compositions makes them potentially advantageous for use in TEVGs.^152,153^ Their inherent mechanical strength reduces the need for *in vitro* culture or may remove this altogether. Suitably decellularized blood vessels possess mechanical properties ideal for use as vascular grafts, and decellularized nonvascular tissue may be conditioned for vascular applications.^147,154^ Additionally, as decellularized matrices are remodeled, they may release chemoattractants with mitogenic or chemotactic activities that stimulate further host cell invasion and assist TEVG integration and remodeling.^155^ However, there are also a number of limitations associated with using decellularized natural matrices. The decellularization process is a compromise between removing antigenic cellular material and maintaining the ECM. Variation between protocols with respect to this balance is large.^156,157^ Inadequate decellularization has been associated with adverse immune reactions and sudden failures in implants, while aggressive treatments may remove important ECM components, such as elastin, leading to altered mechanical properties that may render the tissue no longer fit for purpose.^158–161^ It has also been suggested that *in vivo* recellularization of decellularized tissues may be inhibited by their dense ECM networks restricting cellular invasion or by chemical alterations to the matrix caused by the cell removal processes.^125,162–164^ Indeed, a lack of graft cellularity has been associated with the limited success of the decellularized vascular grafts that have achieved commercial availability. Control over the geometry of decellularized TEVGs is another issue, as the size and shape of the tissue available is restricted. Composite grafts may be constructed, but this adds complexity and expense to the graft manufacturing process and may affect mechanical performance and biocompatibility.^151,152^ Additionally, a number of graft properties, including geometry, mechanical properties, and chemistry, may vary based on the age and health of the donor, making control of graft quality a challenge.

### Tissue engineering by self-assembly

**FIG. 4.**
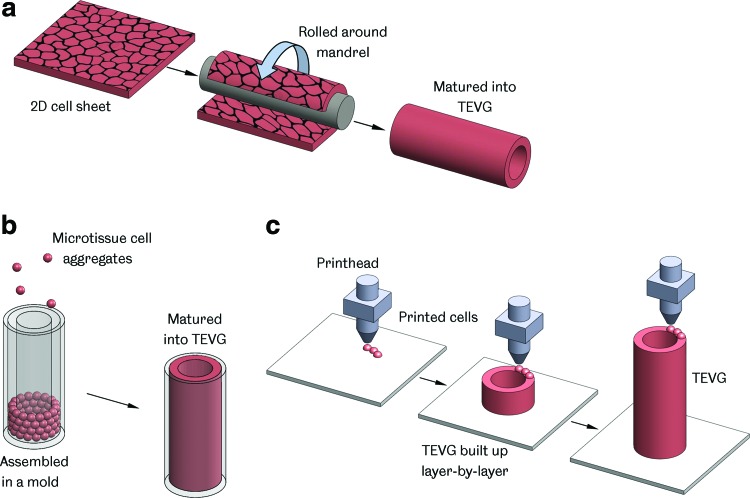
TEVG manufacture by self-assembly. **(a)** Sheet-based tissue engineering—a 2D cell sheet is cultured and then shaped around a mandrel, forming a tube that is matured into a TEVG; **(b)** Assembly of microtissues—cell aggregates placed in a mold and combined to form a TEVG; **(c)** Bioprinting—cells and supporting material are deposited in a layer-by-layer manner, building up a 3D construct. Color images available online at www.liebertpub.com/teb

In a departure from the classic paradigm, tissue engineering by self-assembly (TESA) does not utilize a scaffold or supporting matrix in the creation of a TEVG (Fig. 4). This approach was pioneered in the form of sheet-based tissue engineering, but now includes other methods, such as microtissue aggregation and cell printing.

L'Heureux and colleagues were the first to use sheet-based tissue engineering to produce a TEVG. The process involves the production of sheets of cells, which are then layered and shaped around a mandrel, forming the tubular structure of a vascular graft. Media supplementation is used to encourage the cultured cells to produce large amounts of ECM, thus generating strong and robust sheets for TEVG fabrication.^166^

Early work attempted to produce a TEVG with a structure that mimicked natural arteries.^65^ Sheets of human SMCs were wrapped around a mandrel and followed by sheets of fibroblasts. Culture in a flow bioreactor allowed the layers to fuse together before ECs were seeded onto the vessel's lumen. This process produced a vascular graft with a layered structure similar to natural blood vessels. Although 4 weeks were required to produce the cell sheets and an additional 8 weeks to allow the layers to fuse together, this vessel represented the first TEVG that showed physiologically relevant mechanical properties without the presence of a supporting scaffold. The vessels demonstrated burst pressures of 2594 ± 501 mmHg, well above the human SV. They also displayed physiological behavior, including contractile properties, imparted by the medial SMCs.^167^ *In vivo* studies in canines as femoral artery interposition grafts showed that these grafts could withstand physiological pressures, demonstrating the feasibility of the sheet-based technique.^65^

Further development saw the removal of the medial SMC layer and an evaluation of graft production using age- and risk-matched human cells. Through increased culture times and media optimization, grafts produced from elderly donors with cardiovascular disease achieved the same mechanical strength as those made from young healthy donor cells.^63^ These vessels were evaluated as arterial interposition grafts in nude rats and in a more biomechanically relevant primate model with patency rates of 85%, after up to 225 days, and 100%, after 8 weeks, respectively. The grafts showed good integration with the surrounding tissue and resistance to aneurysm formation, although the overall production times still remained long at up to 28 weeks.

Despite this drawback, the results were encouraging and clinical trials were undertaken with the grafts utilized as AVFs for hemodialysis access.^168^ The TEVGs were produced using autologous fibroblasts and ECs taken from patient biopsies. They ranged between 14 and 40 cm long, with internal diameters of 4.8 mm, and displayed burst pressures of 3512 ± 873 mmHg. Of an original 10 patients, patency rates were 78% at 1 month (*n* = 9) and 60% at 6 months (*n* = 8) (withdrawal from the study and a nongraft-related death reduced the cohort size). Graft failures were associated with thrombosis or aneurysm formation. Over the 20-month trial, four grafts remained patent throughout. The results were in line with the currently set objectives for conventional procedures of 76% patency at 3 months across all populations. However, the study group represented a particularly challenging patient population where AVF failure was expected to be far higher.^169^ With this in mind and the fact that the AVF may be considered a challenging application for a TEVG given the high puncture frequency it is subjected to, the results were considered to be quite promising. A second study examining leg revascularization is now being targeted.^168^

The use of allogeneic cells in sheet-based tissue engineering has subsequently been explored. Grafts produced from allogeneic fibroblasts were devitalized and successfully implanted in three patients to provide hemodialysis access.^170^ Although little immune response was evident, in line with other reports which have shown allogeneic fibroblast constructs to be well tolerated, stenosis which required intervention developed in 2/3 grafts and failures ultimately occurred due to infection or thrombosis at 7 months. Further work is needed to determine if allogeneic cells can be successfully utilized to produce a TEVG, but this study clearly represents an important first step. Additionally, it has been shown that L'Heureux's sheet-based TEVGs can be manufactured and then stored before requirement. A graft was successfully devitalized and stored frozen before being rehydrated, seeded with autologous ECs, and then successfully employed as an AVF in a patient requiring vascular access.^171^ This result, coupled with the potential use of allogeneic cells, offers real potential for a truly off-the-shelf TEVG solution.

Finally, the group of L'Heureux has recently described a new TESA method for producing a TEVG: thread-based tissue engineering. In this study, cell-synthesized threads are produced *in vitro* and assembled into 3D structures using textile techniques such as knitting, braiding, or weaving. Grafts produced using this method have been suggested to possess greater strengths and require shorter production times than their sheet-based equivalents, although detailed reports are still outstanding.^172,173^

Other researchers have also explored the possibilities of sheet-based tissue engineering (Table 5). Grafts produced from sheets derived from mesenchymal stem cells (MSCs) have shown potential in a rabbit model, where they functioned as interposition grafts in the carotid artery for 4 weeks.^174^ The group of Germain used dermal and SV fibroblast cell sheets to generate tubular constructs, which were then decellularized leaving behind only ECM, to act as a scaffold onto which autologous SMCs could be seeded.^175^ Although only currently at the stage of *in vitro* examination, this method offers the potential for an off-the-shelf TEVG solution similar to Niklason's, with the decellularized matrices being produced from allogeneic cells, stored, and then seeded with autologous cells just before implantation.

**Table 5.** Studies Toward the Development of a TEVG Using TESA

*TESA manufacturing method*	*Development level*	*Cell source*	*Comments*	*Group*
Sheet-based tissue engineering	*In vivo* (human trial)	Autologous fibroblasts and ECs	First clinical trial of a TEVG under arterial flow as an AVF. 4/10 grafts patent for up to 20 months (in line with current clinical targets).	L'Heureux and colleagues^63,65,168^
Sheet-based tissue engineering, followed by decellularization	*In vitro*	Human dermal and vein fibroblasts	Decellularized grafts consisted of ECM components only. SMCs proliferated successfully on decellularized grafts.	Germain and colleagues^175^
Mircotissue aggregate assembly	*In vitro*	Human artery fibroblasts and umbilical vein ECs	Cell aggregates bound by secreted ECM assembled into tubes. Fused under dynamic culture to form tube structures.	Hoerstrup and colleagues^176^
Bioprinting	*In vitro*	Human umbilical cord SMCs and dermal fibroblasts	Branched vessel produced from the fusion of printed cell cylinders and spheroids. High cell densities achieved with no scaffold. Maximum burst pressure values of 773 mmHg were achieved after 21 days in bioreactor culture.	Forgacs and colleagues^177,178^

TESA, tissue engineering by self-assembly.

Additionally, novel TESA approaches have recently been reported involving the production of TEVGs by self-assembly of microtissue aggregates.^176^ Using hanging drop cultures of human artery-derived fibroblasts and human umbilical vein endothelial cells, cell aggregates bound by secreted ECM were generated and then assembled into tubes. After 14 days under pulsatile flow, these aggregates had fused into confluent structures. In a similar approach, 3D bioprinting was utilized to produce simple and branched blood vessel constructs by precise deposition and fusion of multicellular spheroids and cylinders.^177^ After 21 days of culture in a bioreactor, these constructs demonstrated burst pressures of 773 mmHg, although this value appeared to have plateaued.^178^ These approaches offer the potential for complex shapes to be produced, such as vascular bifurcations; however, whether they can achieve the required mechanical strength for use in the circulatory system remains to be seen.

TESA sidesteps a number of issues associated with TEVG production using scaffold-based or decellularized matrix methods. Difficulties associated with the manufacture, mechanical properties, or breakdown of these supporting structures are removed. The major limitation of using TESA is the extended *in vitro* culture periods required, with multiple months needed for sheet-based TEVGs to achieve suitable mechanical integrity for vascular applications.^63,168^ This drawback may be circumvented in the future by production in anticipation of the individual's clinical needs, and then storing the vessels until required, or by employing allogeneic cells to yield an off-the-shelf graft solution. Sheet-based tissue engineering is also potentially limited in terms of the geometries it can produce. Thread-based tissue engineering or cell aggregate methods, such as 3D bioprinting, may be more suitable for more complex constructs, but have yet to be proven.

## Outlook—Unanswered Questions and the Future of the TEVG

Despite the vast differences between the approaches being pursued to develop a vascular graft using tissue engineering, a number of similar issues are facing all researchers in this field. These include selecting the most appropriate cell types to use in TEVG production, determining how to achieve and maintain the required graft mechanical properties, understanding the process of TEVG remodeling and integration with the host vasculature, and utilizing the most appropriate animal models for evaluations of potential grafts. These issues must be carefully considered in the future development of this technology as they may hold the keys to the widespread clinical adoption of TEVGs.

### Cell source

A number of different cell types have been used in the *in vitro* preparation of TEVGs (Table 6). The type of cells used may directly affect the structure of the graft and ultimately how it performs *in vivo*, along with impacting the graft manufacturing process.

**Table 6.** Range of Cell Types Employed in TEVG Development

*Cell type*	*Specific cells*	*Advantages*	*Disadvantages*	*Reference*
Autologous Somatic Adult cells	Vascular SMCs Vascular Fibroblasts Vascular ECs Dermal Fibroblasts	Proven by a number of groups in TEVG manufacture. In the case of vascular cells, TEVGs comprise the same cells as native blood vessels.	Harvest of vascular cells is invasive and may be limited by vessel quality or availability. Limited replicative and regenerative potential.	^64,69,72,94,97,100,102,113,120,149,168,177^
Progenitor cells	Bone marrow-derived smooth muscle progenitor cells Vascular EPCs	May be isolated from bone marrow or blood. Compared with adult cells, show greater replicative and regenerative capacity and may be cultured for extended periods to generate more robust TEVGs.	Certain progenitor cells may be depleted in elderly patients.	^84,98,141,142,181^
Natural stem cells	BM-MNCs MSCs Adipose tissue stem cells Muscle-derived stem cells Hair follicle stem cells	Isolated from bone marrow and contain various stem cells. Can generate SMCs, fibroblasts, and ECs. May be extracted from bone marrow, blood, adipose tissue, and liver. Able to differentiate into SMCs. Assist EC colonization of TEVGs. Possess some antithrombogenic qualities. May be isolated from adipose tissue biopsies. Able to differentiate into SMCs and ECs. Patient age appears to have little effect on cell numbers and differentiation potential. Some success shown *in vivo* with seeded TEVGs integrating with the native tissue well. Hair follicles represent an abundant and easily harvested source of stem cells. Can differentiate into SMCs. Greater proliferative potential in culture compared with bone marrow-derived MSCs.	Bone marrow harvest is invasive. Little ability to differentiate into ECs Only early *in vitro* work reported. Muscle biopsies are invasive and painful. Only early *in vitro* work reported.	^54,124,140,184^ ^190,192,193,210,211^ ^194,195,197^ ^74^ ^147,199^
iPSCs	Various adult and embryonic cell sources	Great potential to generate cells for vascular tissue engineering from various adult or embryonic cells.	Differentiated cells produced from iPSCs show varied proliferative potential depending on the original cells used in iPSC generation, highlighting cell source as an important factor.	^204,205^
Nonautologous cells	Allogeneic fibroblasts (many other possible cells yet to be explored)	Time taken to expand patients' own cells in culture avoided. Variation in cell quality between patients avoided. Off-the-shelf grafts possible. A wide variety of cell types, both human and animal, may potentially be used.	Potential immunological issues. Regulatory approval may be challenging.	^173^

EPCs, endothelial progenitor cells; MSC, mesenchymal stem cells; iPSCs, induced pluripotent stem cells.

Autologous adult vascular cells, such as SMCs, ECs, and fibroblasts, have been employed in many cases in creating TEVGs. These cells may be cultured for extended periods in bioreactors or seeded onto grafts before implantation. Despite their popularity, the use of these cells has several drawbacks. Their extraction requires blood vessel biopsies, which are invasive, cause donor site morbidity, and in some cases may be impossible due to vessel quality or availability.^124^ Although some researchers have explored using nonvascular cells in an effort to overcome these issues,^94^ adult cells are also limited in terms of replicative and regenerative capacities due to their age.^179^ This limits *in vitro* culture times, may affect graft performance *in vivo*, and is particularly pronounced in the elderly, who are involved in the majority of revascularizations. Although improvements have been made to the replicative potential of adult vascular cells using gene therapy, little effect on their regenerative properties has been achieved.^179,180^ It has also been shown that extended culture periods and media optimization can allow age- and risk-matched human fibroblasts to produce TEVGs of similar quality to those made from young healthy donor cells, yet such methods may ultimately be impractical.^63^

Given the limitations of autologous adult cells, various stem cell sources have been investigated for vascular tissue engineering. These include (i) progenitor cells; (ii) BM-MNCs; (iii) MSCs; (iv) adipose, (v) muscle, or (vi) hair follicle-derived stem cells; and (vii) induced pluripotent stem cells (iPSCs).

#### Progenitor cells

Compared with adult cells, progenitor cells may be isolated from bone marrow or blood, using far less invasive procedures, and demonstrate greater proliferative and replicative capacities.^98,141,142^ Using these cells may allow for longer *in vitro* culture periods, generating more robust TEVGs.^181^ Ovine bone marrow-derived smooth muscle progenitor cells produced stronger and tougher TEVGs *in vitro* compared with using adult vascular SMCs directly. Progenitor cell-based grafts also produced more organized elastin when implanted *in vivo* as jugular replacements in lambs.^96,98^ Additionally, using vascular EPCs may be advantageous as they induce nitric oxide-mediated vascular relaxation. This process is more pronounced in arteries than veins and has been associated with contributing to the superiority of artery bypass conduits compared with veins.^182^ Vascular EPCs may be depleted in elderly patients, thus making them potentially difficult to obtain.^183^

#### Bone marrow mononuclear cells

BM-MNCs can be extracted from the bone marrow and include MSCs and hematopoietic stem cells. An extract of BM-MNCs has the potential to generate various cell types, including vascular ECs, SMCs, and fibroblasts.^184^ These cells also lack major histocompatibility complexes, along with other important immunostimulatory molecules, offering potential as allogeneic cells for TEVG production.^185^ Shinoka and colleagues employed autologous BM-MNCs, extracted from the superior iliac spine, with great success in their pioneering clinical trial.^54^ Additionally, these cells may be utilized to generate SMCs and ECs for seeding onto TEVGs *in vitro*.^124,140^

#### Mesenchymal stem cells

MSCs may be separated from BM-MNC extracts or other tissues, including blood, adipose tissue, muscle, and liver.^186–189^ They are able to differentiate into SMCs, with *in vitro* studies on TEVGs highlighting the effect that mechanical stimulation and certain growth factors can have on this process.^190^ MSCs have demonstrated little potential to generate ECs, but work in animal models has suggested that they may have a role in assisting EC colonization of TEVGs.^191,192^ Additionally, MSCs may possess antithrombogenic qualities, potentially allowing for their use in TEVGs *in vivo* without the requirement for ECs.^193^

#### Adipose tissue stem cells

Adipose tissue also contains its own stem cells, which have been shown to differentiate into both SMCs and ECs and have been used as a source of cells for vascular tissue engineering.^194–196^ These cells can be extracted in high quantities from adipose tissue aspirate, which is often readily available and easy to harvest. Since the majority of revascularization procedures are conducted on elderly patients, utilizing adipose tissue stem cells may have particular advantages. It has been demonstrated that these cells maintain high potency, with their potential to form ECs appearing unaffected by age.^197^ Additionally, their numbers do not appear to diminish with advancing age with some evidence suggesting that they may actually be more abundant in older subjects.^197,198^

#### Muscle-derived stem cells

Muscle-derived stem cells have been utilized in successful *in vivo* studies of TEVGs.^74^ When seeded onto PEUU scaffolds and implanted in rat aortas, these constructs demonstrated patency for up to 8 weeks, integrated with the surrounding tissue, and became populated with ECs and SMCs. Although these results are positive, muscle-derived stem cells can only be obtained from muscle biopsies. These procedures are invasive and painful and therefore the clinical use of this particular stem cell source may be limited.

#### Hair follicle stem cells

Recently, hair follicle stem cells have been utilized to recellularize SIS and umbilical arteries, following decellularization, to potentially create vascular grafts.^147,199^ Although only early *in vitro* work has been reported, the hair follicle represents an abundant and easily harvested potential source of stem cells for use in TEVG production.^200^ Hair follicle stem cells have been suggested to be similar to MSCs from bone marrow, although with a greater ability to proliferate in culture.^201^ They may also have low immunogenicity, giving them potential as allogeneic cells.^202^

#### Induced pluripotent stem cells

The relatively new discovery of induced pluripotency opens up the possibility of obtaining suitable cells for vascular tissue engineering by transforming adult cells.^203^ In a recent study, murine iPSCs were differentiated into SMC and EC phenotypes and used to construct a TEVG, which remained patent for up to 10 weeks when implanted in the inferior vena cava of a mouse model.^204^ Additionally, human iPSCs, established from vascular fibroblasts, were used to generate proliferative SMCs, which were combined with a PLLA scaffold to create a TEVG that demonstrated vascular tissue formation when implanted subcutaneously in a mouse model.^205^

Although both of these studies are promising and iPSCs have exciting potential for the generation of patient-specific TEVGs, significant knowledge has still to be gained regarding their use. For example, ECs differentiated from iPSCs derived from adult cells have shown reduced proliferation compared with those from embryonic iPSCs, suggesting that the original source for iPSCs may influence the properties of the ultimately derived cells.^206^ Greater understanding of this revolutionary cell type is required and a drive toward achieving this is already clear.^207^

The possibility also exists to employ nonautologous cells in vascular tissue engineering. This could eliminate the problems with cell quality and variation that are associated with patient-specific cells and also remove the delay in graft availability that is often caused by their culture requirements, potentially making grafts available off-the-shelf. Allogeneic cells have been employed successfully in treatments for other tissues, particularly the skin, where allogeneic dermal fibroblasts have been used in approved products, such as Apligraft^®^ and Dermagraft^®^, without immunological issues. Additionally, BM-MNCs and hair follicle stem cells have both been shown to elicit low immune responses in allogeneic applications, thus presenting another possible source of donor cells for TEVGs.^185,202^ L'Heureux and colleagues have trialed a sheet-based TEVG produced from allogeneic fibroblasts in the clinic.^170^ Although patency was limited, an adverse immune response did not appear to be present, suggesting there is more to be learned about utilizing allogeneic cells in vascular graft tissue engineering. It should also be noted, however, that the use of allogeneic ECs in a TEVG is unlikely because of their high immunogenicity.^208^ Future graft designs are likely to remain reliant on autologous ECs isolated from patients.

The range of possible cell types that may be utilized in a TEVG has recently been widened by the use of decellularization protocols for producing engineered vessels *in vitro*, using polymer scaffolds or TESA.^69,175^ With the decellularization process removing the immunogenic cellular material from the grafts, therefore separating the cells used to engineer them from the intended patients, restrictions on cell source become reduced. Allogeneic, or even xenogeneic, banked cells or cell lines could be employed for the *in vitro* culture of the grafts.

The possibility of generating a TEVG without the need for *in vitro* cells is also being explored by a number of research groups. The earliest commercially available vascular grafts based on decellularized tissues did not utilize *in vitro* seeded cells; however, their performance has been limited. No clear advantage of these products over alternative, and less expensive, synthetic conduits has been demonstrated.^135–138^ The complications and failures seen when using these grafts were largely associated with thrombosis and aneurysm, and although the specific mechanisms behind these issues are not fully understood, the lack of graft cellularity has been suggested as a contributory factor.^127,133^ As such, a number of researchers developing decellularized matrix-based TEVGs have now taken to adding *in vitro* cells before implantation. Despite this, research into developing a TEVG that may be acellular at implantation has continued. A number of acellular grafts based on synthetic or natural polymer scaffolds, along with decellularized matrices, have now been explored, although with varied results. Successful cell invasion, remodeling, and integration of acellular polymer grafts have been reported in some *in vivo* studies in rats and canines.^76,78,79^ However, other works have reported poor results with similar grafts showing the development of substantial intimal hyperplasia and calcification or a reduction in integrity after remodeling.^82,122^ Conflict is seen between studies using similar scaffolds, animal models, and timescales, but different implantation sites, suggesting that *in vivo* tissue engineering responses are complex and dependent on as yet unknown factors, such as the hemodynamic environment, inflammation, and the immune response.^80,82^ Additionally, numerous reports have shown failures in acellular grafts, implanted *in vivo*, directly associated with thrombosis due to the lack of a lumenal EC layer.^68,74,124,142,209^ Indeed, platelet adhesion assessments in TEVGs have demonstrated the success of ECs in reducing thrombosis.^122^ However, biochemical surface modification, such as heparin coating, has been suggested as a means to counter thrombosis without the addition of ECs.^125^

Removing *in vitro* cells from the TEVG equation has substantial practical and economic implications, greatly simplifying the pathway to clinical adoption and reducing potential therapy costs. However, it remains to be seen if *in vivo* tissue engineering can be relied on alone, especially in elderly or diseased patients, to generate successful vascular grafts. Developing a clear understanding of the reasons behind the limited performance of those acellular vascular grafts that have been used clinically would be useful in guiding the future development of this technology.

### Mechanical properties

#### Design targets

**FIG. 5.**
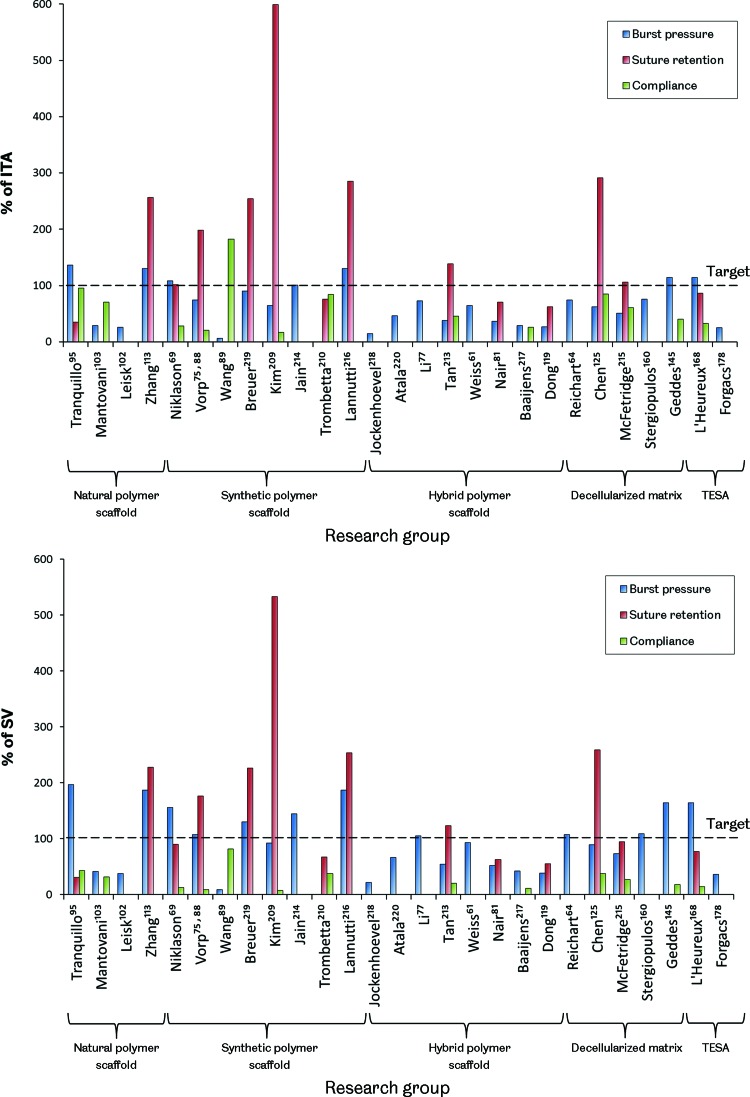
The mechanical properties of reported TEVGs compared with the human internal thoracic artery (ITA) and SV. The results are arranged by manufacturing method and represent grafts before any implantation. For reference, values for burst pressure, suture retention strength, and compliance are 3073 mmHg, 1.72 N, and 11.5%/100 mmHg for the ITA and 2134 mmHg, 1.92 N, and 25.6%/100 mmHg for the SV, respectively.^61–65,210^ Color images available online at www.liebertpub.com/teb

Given the load-bearing nature of blood vessels, resulting from the pressurized fluid flow they support, the mechanical properties of a TEVG are important design requirements. Sufficient mechanical strength to retain integrity and resist permanent deformation may be considered as one of the most fundamental performance criteria. Graft compliance and the way in which deformation under loading occurs are also important as adverse biological responses have been associated with compliance mismatch between native vessels and both synthetic and biological vascular grafts.^32,37^ Additionally, the ability of the graft to retain sutures must also be considered given the surgical methods that will be employed during implantation. There is, however, a lack of agreement over the target values for these particular graft properties among researchers developing TEVGs. It has been common to use the current gold standard graft, the SV, as a target to emulate, making TEVGs essentially SV substitutes. Matching the SV may be beneficial for clinical adoption of a TEVG; however, patency may ultimately be limited by the same mechanical inadequacies associated with the SV. Using arterial conduits in bypass grafting, such as the ITA, has been shown to produce superior patency compared with the SV.^10–13^ These conduits are not used preferentially due to their limited availability and the more severe implications of artery removal compared with veins. Although the ITA may be more challenging to replicate through tissue engineering, due to its increased strength compared with the SV, a TEVG designed to mimic this vessel may also display its improved performance. Figure 5 shows how the mechanical properties of some reported TEVGs compare with both the ITA and the SV. Based on these reported results, it is clear that no TEVG has yet been produced that matches the ITA or SV in terms of vessel burst pressure, suture retention strength, and compliance. Of those groups reporting all of these metrics, the vessels produced by Tranquillo, Niklason, Vorp, Kim, Tan, Chen, McFetridge, and L'Heureux may be tentatively considered the most promising. Interestingly, these groups span the whole range of TEVG manufacturing methods. Clearly, no single method has yet proven its superiority and therefore it is very difficult to suggest the type of TEVG design that may ultimately achieve comparable properties with the ITA or SV.

Another consideration is whether any of the autograft conduits currently used in vascular bypass surgery should be used as a target for the mechanical performance of a TEVG. Since a vascular bypass is a non-natural construct, it is likely that the ideal properties required are different from these vessels. Tissue engineering provides the opportunity to work toward this ideal, but requires additional research to help define any target values. More advanced simulations of vascular biomechanics may be useful in achieving this in the future. Another issue is the variation in blood vessel mechanical properties displayed between different vessels in the human body and between different individuals.^212^ The future of vascular tissue engineering may ultimately involve tailoring specific graft mechanical properties with the intended implantation site. This would require a far greater understanding of vascular biomechanics, the interactions between the graft and the native vessels, and how to engineer the mechanical properties of a TEVG.

#### The effect of remodeling

The picture of TEVG mechanics becomes even more complex when considering the remodeling and alteration in mechanical properties that may occur over time after implantation. It is possible to ask not only what mechanical properties are required but also when are they required. A number of *in vivo* studies have shown that TEVGs remodel to demonstrate altered mechanical properties after implantation. Some cases have shown a negative result, with conduits becoming weaker or stiffer due to an imbalance of ECM deposition and supporting scaffold degradation or calcification.^82,221^ However, positive results with grafts becoming more similar to the native tissue are also reported.^62,69,95,96,145^ The question of how different from the target mechanical performance the graft can be, at implantation, and how quickly it alters *in vivo* is then raised. Given that failures associated with compliance mismatch, such as intimal hyperplasia, may occur in the first year of graft implantation, understanding the process of graft remodeling and the time taken is crucial and requires further investigation.

#### Emulating the native stress–strain response

**FIG. 6.**
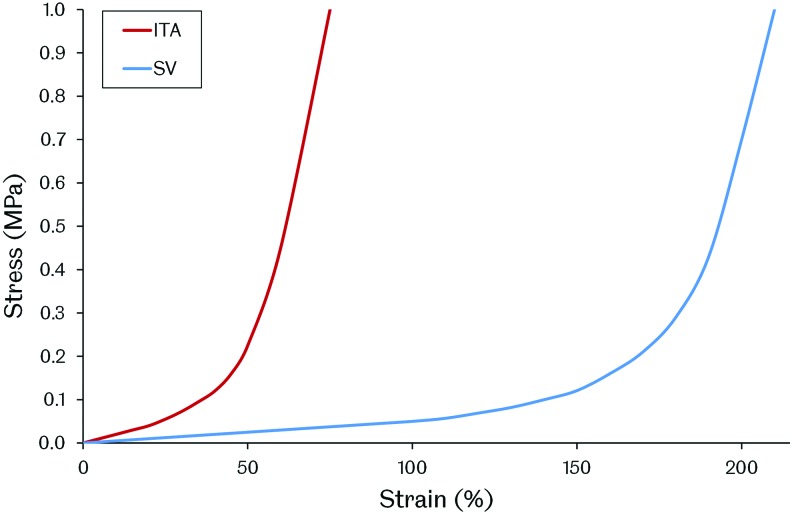
Representative stress–strain responses for the ITA and SV. Both vessels exhibit a J-shaped stress-strain response with a linear toe region. Color images available online at www.liebertpub.com/teb

Emulating the mechanical properties of native blood vessels is a challenge due to their complex behavior. Blood vessels display viscoelasticity and a J-shaped stress–strain response. These properties are a result of the different proteins that form the vessel walls. Low strains produce only small changes in stress driven by the compliant and elastic response of elastin fibers. As strains increase, crimped collagen fibers are opened out and engaged in a tensile manner, causing an increase in stress (Fig. 6).^222–224^

A number of works have reported TEVGs with mechanical responses that are similar to natural blood vessels. TEVGs produced from natural SIS sheets were shown to have a similar, J-shaped stress–strain response to the native ovine carotid artery.^147^ This finding is understandable given their natural soft tissue ECM structure. Using synthetic polymer scaffold-based methods, a PU and polyethylene glycol (PEG)–fibrin hybrid scaffold seeded with mouse smooth muscle progenitors and cultured under pulsatile flow conditions produced a TEVG with a similar stress–strain response to the human coronary artery.^84^ Additionally, a composite vessel produced from layered PCL and PGA sheets seeded with bovine fibroblasts, SMCs, and ECs showed a similar stress–strain response to bovine arteries after dynamic culture for 2 weeks.^91^ *In vivo*, acellular PGS and PCL grafts implanted in Lewis rat aortas remodeled over 90 days, yielding vessels with closely matching mechanical responses to the native vessel.^79^ Mechanical stimulation through pulsatile flow, either *in vitro* or *in vivo*, may have been key in achieving these vessel mechanical behaviors. However, a J-shaped stress–strain response curve similar to the coronary artery was also achieved using an electrospun PEUU scaffold, with integrated rat vascular SMCs, after only 3 days in dynamic culture in a spinner flask.^225^ This result suggests that such pulsatile flow stimulation is not a prerequisite for achieving a TEVG with similar mechanical properties to the native vessel and that scaffold materials may play an important role. Interestingly, an acellular graft produced solely from PGA and PU has also been shown to demonstrate mechanical behavior similar to the natural porcine carotid artery, presenting the possibility of direct implantation *in vivo*, without the need for any *in vitro* cell culture.^214^ It should be noted that it is unclear how the mechanical behavior of TEVGs produced using scaffold-based methods will change over time as the scaffolds degrade. *In vivo* studies of sufficient length and with appropriate time points are required to explore this and capture changes in graft mechanics associated with scaffold breakdown. These studies will contribute to building confidence in long-term graft performance.^78^

#### Elastin

Another interesting element in the endeavour to engineer the required TEVG mechanical properties is elastin production. Elastin fibers are responsible for the elasticity of blood vessels, preventing dynamic tissue creep by stretching under load and returning to their original shape after the load is removed. This property prevents permanent deformation under pulsatile flow.^222^

Although elastin may not be an issue in TEVGs based on decellularized matrices, as these constructs often already possess this protein, producing it in *in vitro*-derived TEVGs is poorly understood. Although their grafts are undergoing clinical trials, the TEVGs produced by Niklason's group have been shown to possess little elastin. This finding has been suggested to be due to acidic hydrolysis products from the breakdown of their graft's PGA scaffolds reducing elastin synthesis by affecting cell proliferation and function, including ECM deposition.^226^ There is some evidence that scaffold stiffness, degradation rate, and topology may influence elastin deposition too.^79,83,85^ Comparing identical PGS and PLGA scaffolds showed that the more elastic PGS produced a TEVG with organized elastin.^85,89^ Further to this, the rapid degradation of a scaffold produced from PGS was also linked to improved elastin deposition in rats.^79^ Organized elastin, in fenestrated sheets, has also been demonstrated when aortic SMCs were cultured on hyaluronan gels.^227^ Additionally, transforming growth factor beta 1 (TGF-β1) was shown to cause increased elastin synthesis in human SMCs only when they were cultured on 3D meshes, not 2D sheets.^83^

Proteins and various factors have also been linked with affecting elastin deposition in TEVGs. Elastin production may be enhanced by fibrin as fibrin gels seeded with rat SMCs showed enhanced elastin production compared with collagen gels.^228^ Ascorbate has been used in the production of TEVGs due to its positive effect on collagen production.^66,69^ However, ascorbate may also inhibit elastin production by destabilizing elastin mRNA.^229^ Using TGF-β1 and insulin was shown to overcome ascorbate's inhibition of elastin and also further enhance collagenesis,^229^ although interestingly Niklason's group used TGF-β1 and ascorbate in the culture of a TEVG and saw little elastin deposition, suggesting that insulin is critical.^190^ Adding further confusion, the groups of Wang and Jockenhoevel both showed elastin deposition when using ascorbate supplementation in the production of TEVGs, therefore its level of elastin inhibition remains unclear.^89,120,218^ Jockenhoevel and colleagues did use a fibrin-containing scaffold, however, suggesting a possible balance between any positive effects of fibrin on elastogenesis and inhibition by ascorbate. Additionally, elastogenesis has been shown to be accelerated by retinoic acid and calcitriol. These compounds are under further exploration to discern their utility in TEVG production.^221,230^

The influence of mechanical stimulation on elastin production in TEVGs is also unclear. Elastin mRNA expression was shown to be independent of mechanical stimulation in collagen scaffolds.^231^ However, in *in vitro* culture in a pulsatile flow bioreactor, a TEVG based on a gelatin–vinyl acetate copolymer scaffold seeded with rat SMCs achieved an elastin content 80% that of the native rat aorta in just 1 week. Although it is unclear how organized this elastin was, gene expression for elastin was upregulated compared with static culture controls.^81^ A synergistic effect between mechanical stimulation and the scaffold material may be in operation. Indeed, it is understandable that the mechanical properties of the scaffold may affect how any mechanical stimulation is transduced onto the cells in a TEVG. Interestingly, in a comparison of PGA and collagen scaffolds cultured under pulsatile flow, elastin expression was only upregulated in the PGA group compared with static controls, suggesting a combinatorial effect of scaffold and mechanical stimulation.^232,233^ Recently, Niklason and colleagues have developed a novel bioreactor able to provide axial and circumferential strain to TEVGs during *in vitro* culture. This biaxial stimulation produced TEVGs with mature elastin fibers, suggesting that axial strain may be another important factor in elastin production.^234^ Elastin deposition has also been shown on SIS sheets cultured *in vitro* under simple uniaxial tension, not pulsatile flow. Human hair follicle-derived SMCs were utilized, but it remains unclear whether these cells, the mechanical properties of the SIS, or its embedded chemical cues may have assisted elastinogenesis.^147^

Evidence for the influence of cell source on elastin production in TEVGs has also been demonstrated. L'Heureux and colleagues were able to achieve elastin deposition *in vitro*, although not quantified, in their sheet-based TEVGs, which are based on fibroblasts, not SMCs, as are commonly used by others.^65^ Additionally, it was shown that using ovine bone marrow-derived smooth muscle progenitor cells compared with using ovine vascular SMCs directly produced more organized elastin in TEVGs implanted as jugular interposition grafts in lambs.^96,98^ Given that elastogenesis varies throughout the mammalian life span, from very high levels during gestation to very low levels in adults, cell age is also likely to be a significant factor in elastin production in a TEVG.^235–238^

TEVGs have also been shown to remodel and gain increased elastin content *in vivo*. A decellularized fibrin scaffold-based TEVG implanted in the ovine femoral artery remodeled to contain 8.8% of the elastin content of the native vessel over 24 weeks, with these fibers being mature and organized. In a canine model, an acellular TEVG placed in the pulmonary artery obtained equal collagen and elastin contents to the native vessel in 12 months.^76^ Additionally, an acellular graft comprising PGA, PCL, PLLA, and collagen implanted as a porcine aorta replacement developed 33% of the native elastin content after 4 months, although elastin production then plateaued.^221^ Determining the process of elastin deposition in graft remodeling may assist in developing methods to modulate and control this or to improve elastin production in TEVGs *in vitro*.

Understanding how to control elastin production is a key challenge in engineering TEVG mechanical properties. In the meantime, it may be possible to circumvent the need to generate elastin by adding it directly. Elastin has been successfully added to scaffolds by electrospinning.^220^ Indeed, an electrospun PCL and elastin scaffold has demonstrated mechanical properties similar to the ITA and was patent in rabbits for 1 month when implanted in an acellular state.^61^

### TEVG hemodynamics

Vascular bypass hemodynamics has been identified as having an effect on graft patency. In particular, intimal hyperplasia formation around the distal anastomosis has been linked with certain blood flow characteristics, such as flow separation, wall shear stress gradients, and flow oscillation or stagnation.^33,239–241^ The occurrence of these undesirable hemodynamics has been suggested to be influenced by both anastomosis geometry and graft compliance, although the former appears to have a greater effect.^242,243^ To this end, a number of different anastomotic configurations have been explored, using both synthetic and autograft conduits, in an attempt to reduce undesirable hemodynamics and improve long-term graft patency. Interposition vein cuffs, such as the Miller cuff and Taylor Patch, improve graft–host compliance matching and also alter anastomotic hemodynamics.^244–246^ Their effectiveness is questionable, however, with conflicting reports of improved patency restricting their wide-scale adoption.^247–250^ Additionally, precuffed synthetic grafts are also in production, again with the intention to reduce undesirable hemodynamics; however, their effectiveness is also debated.^251–253^

More complex anastomotic configurations have been designed and explored through simulation work.^254–257^ Variation in design parameters, such as anastomosis angle, flow area, bypass plane, and graft–host ratio, has been investigated in an effort to define the ideal geometry in terms of blood flow characteristics.^258–261^ It is noted in a number of these works that the designs may be too complex for surgeons to reproduce using current vascular conduits. This difficulty presents a clear opportunity for the TEVG. A tissue-engineered vessel may allow for more complex anastomotic configurations to be created and explored. Scaffold or self-assembly-based approaches to generating TEVGs could be used to construct more complex vascular grafts with the intention to reduce undesirable hemodynamics and improve overall patency. Computer modeling with computational fluid dynamics and finite element analysis could be utilized to develop optimal graft designs and inform vascular graft tissue engineering.^37,49,262,263^ Simulation work is ideal for exploring complex flow parameters, which can be difficult to measure *in vivo*, and also offers high resolution, repeatability, and the option to easily change model settings and explore different graft geometry and flow scenarios. It remains for such a strategy to be examined by researchers in the field of vascular graft tissue engineering, but the potential power of this approach should not be overlooked.

It is also important to note that graft hemodynamics may also have an influence on TEVG integration and remodeling. Indeed, the difference between the blood flow in the carotid artery and the aorta was suggested to be the reason for the largely different calcification and graft cellularization observed between two similar electrospun PCL grafts implanted in an acellular state in a rat model.^80,82^ The role that hemodynamics may play in graft integration remains to be determined. It is possible that as our understanding grows, the design of TEVGs in the future may involve consideration of conduit hemodynamics with relation to both graft integration and remodeling along with the suppression of undesirable flow characteristics associated with causing intimal thickening.

### TEVG remodeling and integration *in vivo*

Although a number of TEVGs have been implanted *in vivo*, in humans and in a number of animal models, the mechanism by which these grafts integrate into the host's circulatory system and remodel into functional blood vessels is largely unclear. Evidence suggests that the host's immune cells, particularly monocytes, macrophages, and neutrophils, may be the major mediators of graft remodeling and neotissue formation through a modified inflammatory response. Immediately after TEVG implantation, neutrophils and monocytes migrate into the anastomosis and an inflammatory response occurs with the removal of debris, resulting from the surgical trauma, by phagocytosis. This process may last several weeks. Subsequently, signals are produced to direct a shift from inflammation to tissue remodeling and repair. Tissue-engineered graft integration appears to consist of an atypical response to vascular injury, including intimal thickening and neointima development, along with biomaterial-related effects in some cases, such as foreign body reaction, fibrosis, and the formation of vascular media.^264^

Monocytes may be particularly important in early graft remodeling. These cells may be attracted to the implantation site due to chemoattractants released by activated neutrophils.^265^ In studies using polymer scaffold-based TEVGs, it has been shown that monocytes may remain at the implantation site until the scaffold is fully degraded (up to 100 weeks has been observed) with localization around residual polymer fragments.^73^ This finding suggests that they may be involved in the complete remodeling process. Monocytes may produce cytokines, growth factors, and enzymes important for vascular cell proliferation and tissue remodeling, such as interleukin-6 and -10 and matrix metalloproteases.^266^ Additionally, macrophage invasion has been observed in a number of acellular TEVGs when implanted *in vivo*.^79,82,95,219^ These macrophages may be derived from monocytes attracted to the implant site and have been suggested to be critical for neovessel formation in a mouse model, although the method by which this occurs is unclear.^267^ The role of different macrophage phenotypes in biomaterial integration has become clear over recent years and is reviewed more thoroughly elsewhere.^268^

Progenitor cells have also been suggested as having an important role in TEVG integration. There is evidence that circulating progenitor cells from the bone marrow contribute to TEVG colonization. When acellular, silk fibroin scaffolds were implanted as aorta replacements in transgenic rats, with bone marrow cells modified to express green fluorescent protein (GFP), GFP-positive SMCs were found as a major cellular component of the graft's medial layer after 12 weeks.^101^ Circulating progenitor cells were also suggested to be the source of the intimal cells that invaded an acellular PGA and PLLA graft implanted in the carotid artery of a canine model.^78^ Evidence suggests that invasion of TEVGs by host cells occurs at the anastomosis, and not through the graft lumen.^141^ It is possible that the host's immune cells around the anastomosis may modulate this process by recruiting circulating progenitors. Interestingly, there is evidence that monocytes themselves may give rise to EPCs.^269^ These cells have been shown to be important in graft endothelialization and thus may represent an additional method by which monocytes contribute to graft remodeling.

Another important question relating to TEVG remodeling and integration is the role of any seeded cells present on the implanted graft. There is evidence that cells present on TEVGs at the time of implantation are quickly lost or replaced by the host's own. GFP labeling of ECs seeded onto a decellularized TEVG showed that most were lost and replaced by host cells after 30 days *in vivo* in a porcine model.^68^ Another study showed that only 10% of seeded EPCs remained present on a decellularized graft, after 130 days, implanted in the ovine carotid artery.^142^ Additionally, studies in rats with PEUU grafts seeded with human pericytes found that most were lost from the graft after 8 weeks and replaced by the host ECs and SMCs.^75^ It is unclear how cells present on TEVGs at implantation may interact with the host and influence graft remodeling, although this process is likely to be dependent on the specific cells involved. Interestingly, human BM-MNCs seeded onto polymer scaffolds were found to be replaced by monocytes within just a few days of *in vivo* circulation in a mouse model. These monocytes were later replaced by SMCs and ECs. It was noted that the BM-MNCs secreted significant amounts of monocyte chemoattractant protein 1 (MCP-1) as a result of their contact with the polymer scaffold. This may have contributed to monocyte recruitment.^270^ It may be possible to exploit this process and encourage monocyte invasion into TEVGs by using MCP-1. Polymer scaffolds have been created with bound MCP-1 releasing microparticles in an effort to achieve this.^270^

Although the cellular and molecular mechanisms of graft integration and remodeling remain largely unclear, gaining understanding in this field is vital for the future development of TEVGs. With additional knowledge, it may ultimately allow modulation and engineering of this process and enhanced implant integration.

### Animal models and *in vivo* studies of TEVGs

A number of different animal models have been used to examine TEVGs *in vivo* (Table 7). No single model is optimal for studying all the performance criteria associated with a TEVG, such as implantability, mechanical performance, biocompatibility, thrombogenicity, and hemodynamics. Therefore, selection must be made carefully and with the particular criteria under investigation in mind.^271–273^ Considering the implantation site, vessel diameter and anastomosis are important for assessing TEVG hemodynamics, intimal hyperplasia, and implantability. Similarity to the human circulatory system is key for assessing graft mechanical performance, along with thrombosis and integration; the latter could involve consideration of animal age also given the reduced regenerative powers of older cells.^82^ Additionally, longer grafts should be selected for longer-term patency assessments. These considerations must be weighed against model specific factors, such as animal availability; ease of handling; ease of performing the implantation surgery; study duration; compatible methods of graft analysis, such as imaging techniques, and cost.

**Table 7.** Animal Models Available for Studying TEVGs *In Vivo*

*Animal model*	*Advantages*	*Disadvantages*
Rat and Mouse	Low cost allows for large sample size. Wide variety of transgenic lines available, allowing exploration of genetic/molecular mechanisms affecting TEVG implantation. Ideal for biocompatibility and cell infiltration studies.	Limited to short-term studies due to dissimilarity to the human circulatory system. Only very small grafts (<2 mm diameter, 10 mm long) can be examined. Thrombogenicity mechanisms are not similar to humans.
Rabbit	Small in size, but possess greater similarity to human physiology than rats and mice. Can accept clinically relevant graft sizes (1–4 mm diameters). Endothelialization rates and thrombogenicity mechanisms similar to humans. Multiple implantation sites available (aorta, carotid, or femoral arteries).	Limited to short-term studies due to animal size and vascular physiology.
Canine	Multiple implantation sites available, including large vessels (aorta and thoracic artery) and small vessels (carotid and aortoiliac arteries). Lack of spontaneous endothelialization can provide a more stringent environment for TEVG assessment.	Thrombogenicity mechanisms significantly differ from humans. Vessel viscoelastic properties differ from humans. Immune response restricts study lengths.
Pig	Similar vascular physiology and anatomy to humans. Well established as a model for assessing cardiovascular medical devices. Can be used to assess plaque formation in short-term studies. Smaller-sized versions (miniature pigs) are also available, which are easier to handle.	Rapid animal growth presents difficulties in handling. Mount an extensive immune response to implanted tissues. Studies may be limited to shorter terms compared with other large animals.
Sheep	Cardiovascular physiology and thrombogenicity mechanisms similar to humans. Suitable for testing clinically relevant graft sizes (4–6 mm diameters). Long-term studies possible. Natural long neck allows easy implantation into the carotid artery and monitoring through noninvasive techniques, such as ultrasound. Endothelialization mechanisms similar to humans.	Tendency to hypercoagulability.
Nonhuman primate	Greatest similarity to human physiology and cardiovascular anatomy of any animal model. Thrombogenicity and atherosclerosis mechanisms similar to humans. Multiple, clinically relevant implantation sites available. Compatible with a wide range of noninvasive imaging techniques adapted from humans.	High cost. Ethical concerns associated with using primates in medical research.

Rats and mice have been used extensively in TEVG development. Their low cost and ease of care allows for increased sample sizes to be evaluated and the availability of a variety of transgenic strains makes them useful for examining the molecular mechanisms involved in TEVG remodeling.^267^ However, the dissimilarity of their circulatory system to that of humans limits their utility to short-term studies of only very small grafts (<2 mm diameter). Rabbits are also suitable for TEVG assessments, but have been relatively underutilized. Although also limited to short-term studies, they possess similarity to human physiology, in terms of endothelialization rates and thrombogenicity, and are able to accept clinically relevant graft diameters (1–4 mm). Canines differ from humans in terms of thrombogenicity and represent a more stringent model for TEVG investigations due to their lack of spontaneous endothelialization. They provide a range of implantation site options with varied vessel sizes, although their immune response restricts study lengths. Pigs and sheep both show similarities to human physiology. Pigs may be used to assess TEVG growth and plaque formation over the short term, while sheep are suitable for studying thrombogenicity, calcification, and long-term graft patency.^274^ Compared with dogs, pigs, or sheep, nonhuman primates, such as baboons and macaques, possess the cardiovascular physiology most similar to humans. They also have comparable thrombogenicity mechanisms and are susceptible to atherosclerosis formation. Additionally, their physical similarities make them compatible with a number of imaging techniques and testing assays developed in human medicine. Despite these advantages, nonhuman primates have been used rarely in TEVG evaluation due to their high cost and the ethical issues they also raise.^63,69^

## Conclusions

It is clear that a number of different approaches are being explored to produce a TEVG and, with clinical results being reported for a range of techniques, the best solution is yet to be determined. Despite the range of technologies, all apparently poised for success in this field, it is clear that our understanding of what is required from a TEVG, how to create the ideal graft, and how to direct graft integration once implanted must be improved.

A greater understanding of the biomechanical properties for the ideal TEVG is required, with data from simulation work and *in vivo* trials needed to provide this. Additionally, knowledge of how TEVGs integrate with the host after implantation and remodel into a part of the vascular network is crucial to allow control of this process.

**FIG. 7.**
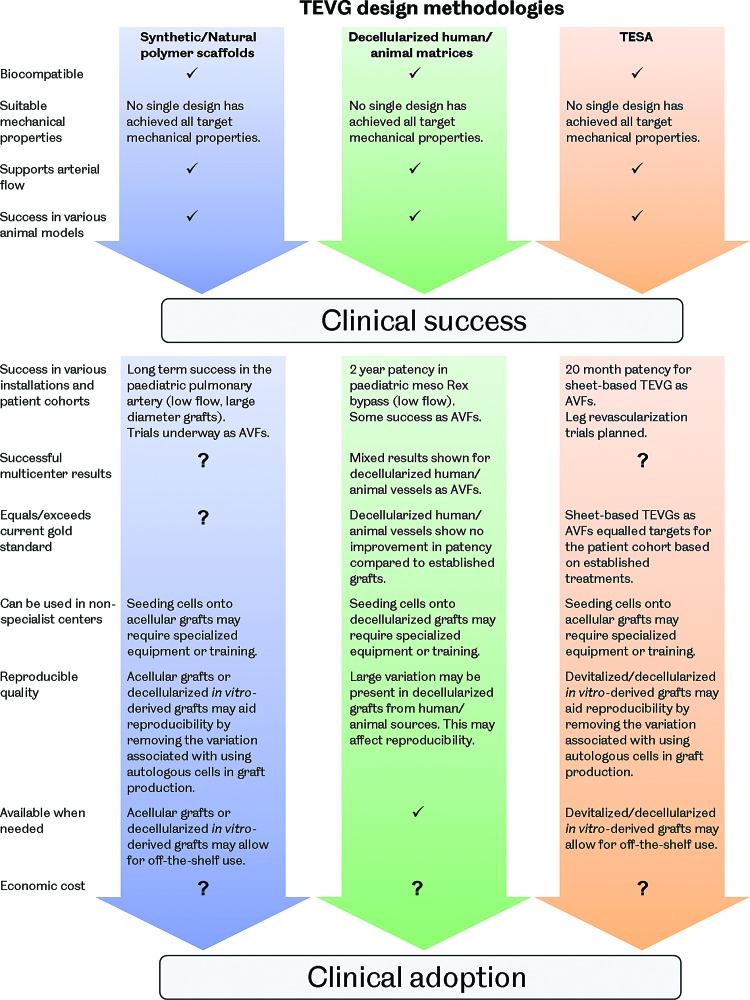
Pathway for the development of the TEVG from design concept to clinical success and then clinical adoption. The various criteria for achieving these milestones are detailed and discussed in relation to the three major TEVG design methodologies. Color images available online at www.liebertpub.com/teb

It is likely that a combination of both clinical results and economic considerations will be the driving force in determining which of the various approaches to TEVG production ultimately see wide-scale adoption (Fig. 7). Even with successes in patients, all developers will need to navigate the pitfalls of bringing a tissue-engineered product to market. When considering the varied regulatory environments and healthcare economics of different nations, this is a considerably complex task.^275,276^ Indeed, commercialization of tissue-engineered products has proven to be particularly difficult in the past, although the tissue engineering industry as a whole is now showing clear movements toward becoming profitable.^277,278^ Evaluation of the healthcare market and targeting the most appropriate vascular graft procedures where TEVGs could be beneficially employed will be important in the early years of commercial use and assist in establishing the technology. A recent study suggested that even though CABG procedures are more common, peripheral artery revascularizations and AVFs for vascular access demonstrated stronger commercial feasibility as the initial target market for the TEVG in the clinic.^279^ It is also likely that in the future of healthcare, cost/benefit will not be the only justification for a new medical technology such as the TEVG. Demonstrating value to the patient and medical system is also likely to become a more important factor.^280^

A clear shift to greater consideration of the clinical use of TEVGs, in terms of both practicality and commercial potential, is evident from the recent developments in attempting to create off-the-shelf solutions reported by a number of groups. Grafts that are readily available to patients are more attractive to clinicians and offer greater commercial viability.^275^ This demonstrates a clear change in outlook by researchers in TEVG development toward technology translation.

The opinions of clinicians and vascular surgeons will also be an important factor as TEVGs move into clinical use. These groups may present a significant obstacle as persuading a shift from the well-established current practices to the new approaches tissue engineering offers may be difficult without significant clinical data to support adoption. These may take a number of clinical trials and years to gather.

The development of the TEVG is at a critical point. The future appears positive with a number of diverse technologies, all competing toward the same goal and all showing the potential for success. Which will emerge as clinical products is unclear. What is clear is the substantial research effort driving toward this and the great clinical impact this particular branch of tissue engineering technology could have as it transitions from medical research to medical practice.
